# Broad Purpose Vector for Site-Directed Insertional Mutagenesis in *Bifidobacterium breve*

**DOI:** 10.3389/fmicb.2021.636822

**Published:** 2021-03-23

**Authors:** Emily C. Hoedt, Francesca Bottacini, Nora Cash, Roger S. Bongers, Kees van Limpt, Kaouther Ben Amor, Jan Knol, John MacSharry, Douwe van Sinderen

**Affiliations:** ^1^APC Microbiome Ireland, University College Cork, Cork, Ireland; ^2^NHMRC Centre of Research Excellence in Digestive Health, School of Medicine and Public Health, The University of Newcastle, Callaghan, NSW, Australia; ^3^Department of Biological Sciences, Munster Technological University, Cork, Ireland; ^4^Danone Nutricia Research, Utrecht, Netherlands; ^5^Laboratory of Microbiology, Wageningen University, Wageningen, Netherlands; ^6^School of Microbiology, University College Cork, Cork, Ireland; ^7^School of Medicine, University College Cork, Cork, Ireland

**Keywords:** bifidobacteria, functional genomics, mutagenesis, DNA methylation, synthetic vector

## Abstract

Members of the genus *Bifidobacterium* are notoriously recalcitrant to genetic manipulation due to their extensive and variable repertoire of Restriction-Modification (R-M) systems. Non-replicating plasmids are currently employed to achieve insertional mutagenesis in *Bifidobacterium*. One of the limitations of using such insertion vectors is the presence within their sequence of various restriction sites, making them sensitive to the activity of endogenous restriction endonucleases encoded by the target strain. For this reason, vectors have been developed with the aim of methylating and protecting the vector using a methylase-positive *Escherichia coli* strain, in some cases containing a cloned bifidobacterial methylase. Here, we present a mutagenesis approach based on a modified and synthetically produced version of the suicide vector pORI28 (named pFREM28), where all known restriction sites targeted by *Bifidobacterium breve* R-M systems were removed by base substitution (thus preserving the codon usage). After validating the integrity of the erythromycin marker, the vector was successfully employed to target an α-galactosidase gene responsible for raffinose metabolism, an alcohol dehydrogenase gene responsible for mannitol utilization and a gene encoding a priming glycosyltransferase responsible for exopolysaccharides (EPS) production in *B. breve*. The advantage of using this modified approach is the reduction of the amount of time, effort and resources required to generate site-directed mutants in *B. breve* and a similar approach may be employed to target other (*bifido)bacterial* species.

## Introduction

Bifidobacteria are common gut commensals that have been reported to elicit a number of beneficial effects on their host (Tojo et al., 2014; O’Callaghan and van Sinderen, 2016; Wong et al., 2019). These Gram-positive obligate anaerobes provide the host with nutrients through the breakdown of indigestible dietary carbohydrates (Turroni et al., 2018), have been shown to modulate the immune system (O’Hara and Shanahan, 2007), alleviate symptoms in IBS (O’Mahony et al., 2005), and assist with pathogen exclusion (Lee and O’Sullivan, 2010). However, the precise mechanism of action for most of these attributes is still unclear. Site-directed gene disruption methods involving the use of non-replicating insertion plasmids [e.g., the insertion vector Ori^+^ RepA^–^ pORI28, originally developed for use in *Lactococcus lactis* (Law et al., 1995; Leenhouts et al., 1996)] are currently employed to understand the function of target genes. Notably, the lack of a replication (*repA*) gene within the pORI28 sequence requires the use of a RepA^+^ helper strain to supply the RepA protein *in trans* to allow plasmid replication and maintenance (Law et al., 1995; Leenhouts et al., 1996). Members of *Bifidobacterium* are notoriously recalcitrant to genetic manipulation due to their thick cell wall, sensitivity to oxygen, and extensive and diverse Restriction-Modification (R-M) systems encoded within their genome sequence (Brancaccio et al., 2013; Bottacini et al., 2018b). As a result, one of the major limitations in using commonly available insertion vectors for targeted mutagenesis is the presence of various restriction sites, making them sensitive to the activity of endogenous restriction endonucleases encoded by the target strain (O’Connell Motherway et al., 2009; Bottacini et al., 2018b). Despite all of these difficulties, targeted mutants have been successfully made in a limited number of strains (Hirayama et al., 2012; Sakaguchi et al., 2012; Wei et al., 2012; Hidalgo-Cantabrana et al., 2015; O’Callaghan et al., 2015). For instance, a number of genes in *Bifidobacterium breve* UCC2003 have been knocked-out employing the insertion vector Ori^+^ RepA^–^ pORI19-tet (O’Connell Motherway et al., 2009), a derivative of the pORI28 system containing a tetracycline selection marker for *Bifidobacterium*. However, in order to facilitate the introduction of pORI19-tet into a target strain at sufficiently high frequency to allow gene disruption by homologous recombination an additional step of methylation of the vector is required. This methylation can be achieved in two ways: (i) the plasmid construct is introduced into a methylase positive *Escherichia coli* strain (e.g., the DAM^+^ *E. coli* EC101) which may express a cloned methylase from an active R-M system present in the bifidobacterial target strain; (ii) the less frequently employed chemical methylation. This multi-step approach, despite being successfully applied previously, presents some practical hurdles and limitations: first of all the intermediate step of methylase cloning and methylation of the vector in *E. coli* is quite laborious and time consuming, secondly the system may not always be applicable in cases where target strains contain multiple active R-M systems or in cases where there is no information available on the R-M systems of the target strain.

There are three types of base modification within bifidobacteria and can be detected using a combination of PacBio SMRT and Illumina bisulfite sequencing (BS-seq) (Darst et al., 2010; Bottacini et al., 2018b), these consist of N6-methyladenine (m6A), N4-methylcytosine (m4C) and 5-methylcytosine (m5C). Previous work by O’Callaghan et al. (2015) showed how Pacbio sequencing and methylome analysis of two *B. longum* subsp. *longum* strains (NCIMB 8809 and CCUG 30698) has allowed the construction and synthesis of a tetracycline resistance gene (*tetW*), previously identified in *B. longum* H66 (Flórez et al., 2006), free of *Eco*RII restriction sites. The cloning of this bifidobacterial *tetW* gene into the Ori^+^ RepA^–^ pORI19 system coupled with the use of an *E. coli-Bifidobacterium* shuttle vector expressing a *B. longum* methyltransferase (MTase) from an active R-M system present in the target strain increased the accessibility for genetic manipulation (O’Callaghan et al., 2015). In a later study by Bottacini et al. (2018b), PacBio sequencing was used to compile a catalog of R-M systems encoded by *B. breve* strains. By employing a combination of PacBio sequencing (to predict m6A and m4C methylated bases) and bisulfite-treated Illumina sequencing (to detect m5C-methylated bases) the authors obtained a clear evaluation of the genetic barriers imposed by R-M systems within the *B. breve* species.

In the current study, we present an adapted method for bifidobacterial targeted mutagenesis based on a synthetically engineered derivative of pORI28 (henceforth referred to as pFREM28), from which all R-M motifs as previously identified in *B. breve* are removed and in which the original resistance marker is substituted with a bifidobacterial erythromycin resistance gene. The functionality of this system was validated for three distinct *B. breve* strains (UCC2003, NRBB01, and NRBB57), in which we successfully performed site-directed mutagenesis. The synthetic insertion vector pFREM28 represents a novel application of methylome data to circumvent the requirement of plasmid methylation for site-directed mutagenesis in *B. breve*. The pFREM28 vector requires no methylation before electroporation into the target strains, and its successful application implies that there is further potential for this approach to be applied for the design of custom-made synthetic plasmids to target other “genetically recalcitrant” *Bifidobacterium* species.

## Materials and Methods

### Bacterial Strains and Routine Culture Conditions

*Escherichia coli* and bifidobacterial strains used in this study are detailed in Table 3. *E. coli* was routinely cultured in Luria Broth/agar (LB; 10 g/L tryptone, 5 g/L yeast extract, and 10 g/L sodium chloride, and where appropriate 20 g/L agar) aerobically at 37°C and broth cultures shaken at 180 rpm. Bifidobacterial cultures were routinely cultivated in Reinforced Clostridial Medium/Agar (RCM/A; Oxoid Ltd., United Kingdom). Where necessary, modified de-Man-Rogosa-Sharpe (mMRS) Medium was used of the following composition: 10 g/L Tryptone (Peptone from Casein), 2.5 g/L yeast extract, 3 g/L tryptose, 3 g/L potassium phosphate dibasic (K_2_HPO_4_), 3 g/L potassium phosphate monobasic (KH_2_PO_4_), 2 g/L tri-ammonium citrate, 0.2 g/L pyruvic acid (sodium pyruvate), 0.575 g/L magnesium sulfate heptahydrate (MgSO_4_.7H_2_O), 0.12 g/L manganese (II) sulfate tetrahydrate (MnSO_4_.4H_2_O), 0.034 g/L iron (II) sulfate heptahydrate (FeSO_4_.7H_2_O), 1 mL/L Tween80, broth supplemented with 0.05% L-cysteine-HCL. Bifidobacterial cultures were incubated at 37°C under anaerobic conditions in an anaerobic chamber (10% H_2_, 10% CO_2_, and 80% N_2_). BioMérieux ETEST^®^ (bioMérieux, France) strips for chloramphenicol and erythromycin were used to assess minimum inhibitory concentrations (MIC) for each strain, bifidobacterial strains were on RCA plates incubated anaerobically for 24 h at 37°C and *E. coli* on LB agar. Where appropriate growth media contained chloramphenicol (Cm; 5 μg ml^–1^ applicable for all bifidobacterial strains described here), or erythromycin (Em; *E. coli* EC101 200 μg ml^–1^, UCC2003 150 μg ml^–1^, or 5 μg ml^–1^ for NRBB01/57), which were used for selection of *E. coli* or *B. breve* transformants.

### *In silico* Design of pFREM28 Vector and Plasmid DNA Preparation

Restriction-Modification motifs for all current *B. breve* strains (including NRBB01, NRBB57, and UCC2003) had previously been identified (O’Connell Motherway et al., 2009; Bottacini et al., 2018b) using a combination of SMRT/bisulfite sequencing and comparative genome analysis (Figure 2B). Using an *in silico* method, the sequence of the suicide plasmid pORI28 was retrieved online^1^ and used as a template, the native promoter and erythromycin resistance marker were replaced *in silico* with the sequence of an alternative Em^R^ marker capable of efficient expression in *B. breve* (locus_tag NRBB51_1114) (Bottacini et al., 2018a). Upstream of the coding sequence Em^R^ marker, a sequence of lactococcal P44 promoter (van der Vossen et al., 1987) was introduced *in silico*. All the sequence editing and removal of restriction sites were performed manually using the SnapGene v2.3^2^ and Artemis (Carver et al., 2012) software tools before synthesis. Finally, BLASTP alignment was used to ensure the preservation of the sequence identity of the Em^R^ antibiotic selection marker (Bottacini et al., 2018b) after the introduced base substitutions. The resulting *in silico* constructed vector, which was designated pFREM28, was sent for synthesis using a commercial DNA synthesis provider (performed by BASECLEAR, Netherlands), and the obtained sequence was validated and delivered by this provider as a cloned fragment in the *E. coli* vector pUC57. Unique restriction sites (*Xba*I) were included at the left and right end of pFREM28 to allow for excision from pUC57 by restriction digestion. Following self-ligation and circularization of the obtained pFREM28 vector, the conditional replication functionality (Figure 2A) was confirmed using *E. coli* strain EC101, also demonstrating that the erythromycin MIC was > 256 μg/mL in this strain. Due to the medium/low copy number of this plasmid all subsequent plasmid work described is conducted using plasmid DNA extracted using GeneJET Plasmid Maxiprep Kit (Thermo Scientific) following the manufacturer’s instructions coupled with an ethanol precipitation to concentrate the DNA.

### Insertion Mutagenesis Plasmid Preparation

Targets for insertion mutagenesis were chosen and where applicable homologous regions were selected for amplification with Q5^®^ High-Fidelity DNA Polymerase (BioLabs). Primers included restriction sites (Table 2), not present within the target gene, and allowed cloning of the insertion amplicon into the multiple cloning site of pFREM28. Restriction and ligation of the insertion amplicon and pFREM28 was completed following manufacturer’s instructions and each clean-up step consisted of ethanol precipitation. Ligations were transformed into EC101 competent cells as described previously (Law et al., 1995) and plated on LB agar with 200 μg/mL erythromycin. Sequence validated clones were then recovered as described above.

### Preparation of Electrocompetent Cells

Strains were cultivated overnight at 37°C in an anaerobic chamber (10% H_2_, 10% CO_2_, and 80% N_2_) using either autoclaved or filter (0.2 μM) sterilized mMRS, supplemented with 0.05% L-cysteine⋅HCl. A selection of filter sterilized (0.2 μM) carbohydrates (glucose, lactose, fucose, Lacto-*N*-Neotetraose (LNnT) or lactose + fucose- 0.01 g/mL final) were tested to ascertain their impact on the transformation efficiency of each strain and as a result glucose was used to supplement mMRS for UCC2003 and NRBB57, while fucose + lactose was the carbohydrate combination used for NRBB01-associated transformations (0.01 g/mL final). Fresh 40 mL of mMRS was then inoculated with 5 mL of the overnight culture and 5 mL of select carbohydrates (0.01 g/mL final) and grown to an OD_600_ of 0.6. Cultures were then incubated on ice for 20 min and subsequently centrifuged (4,052 × *g*, 10 min at 4°C). Cell pellets were then washed twice with ice cold sucrose-citrate buffer (0.5 M sucrose and 1 mM ammonium citrate, pH 5.8) before resuspension in 200 μL of the same wash buffer. Electro-transformations (25 μF and 200 Ohms) with varied voltage (1,500, 1,750, 2,000, 2,250, and 2,500 V) were assessed (optimal: 25 μF, 200 Ohms, 2,500 V) with 50 μL of this suspension. Plasmids pNZ44 and pNZ123 (Table 3) were used in preliminarily tests at increasing concentrations (0.1, 0.2, 0.3, 0.5, 1, and 3 μg plasmid DNA) to determine the optimal concentration for maximal transformation efficiency of pFREM28 constructs (optimal: 3 μg). Electrotransformations were conducted using 2 mm electroporation cuvettes. After transformation, the cells were suspended in 1 mL of RCM and incubated anaerobically for 1 h at 37°C. Serial dilutions were plated on RCA containing erythromycin (5 μg/mL for NRBB01 and NRBB57, and 150 μg/mL for UCC2003) and plates were incubated anaerobically at 37°C for 48 h after which time the number of transformants were enumerated. Summary of the original and optimal transformation conditions are outlined in Table 1.

**TABLE 1:** Original and optimal transformation conditions for UCC2003, NRBB01, and NRBB57.

Strain	COH	Media preparation	Aerobic vs. anaerobic	Amount plasmid DNA	Plasmid	Voltage	Transformation efficiency (transformants/μg DNA)
**Original “standard” transformation parameters**							
UCC2003	Glucose	Autoclaved	Aerobic	200 ng	pNZ44	2,000 V	1.67 × 10^3^ ± 2.33
NRBB01	Glucose	Autoclaved	Aerobic	200 ng	pNZ44	2,000 V	1.25 × 10^3^ ± 1.37
NRBB57	Glucose	Autoclaved	Aerobic	200 ng	pNZ44	2,000 V	9.89 × 10^2^ ± 6.34
UCC2003	Glucose	Autoclaved	Aerobic	200 ng	pNZ123	2,000 V	2.00 × 10^7^ ± 2.11
NRBB01	Glucose	Autoclaved	Aerobic	200 ng	pNZ123	2,000 V	8 × 10^6^ ± 5.65
NRBB57	Glucose	Autoclaved	Aerobic	200 ng	pNZ123	2,000 V	4.7 × 10^5^ ± 5.24
**Optimal transformation parameters**							
UCC2003	Glucose	Filtered	Aerobic	3 μg	pNZ44	2,500 V	6.16 × 10^3^ ± 4.37
NRBB01	Fucose + lactose	Filtered	Aerobic	3 μg	pNZ44	2,500 V	3.93 × 10^3^ ± 0.51
NRBB57	Glucose	Filtered	Aerobic	3 μg	pNZ44	2,500 V	5.60 × 10^4^ ± 1.59
UCC2003	Glucose	Filtered	Aerobic	3 μg	pNZ123	2,500 V	1.5 × 10^7^ ± 1.40
NRBB01	Fucose + lactose	Filtered	Aerobic	3 μg	pNZ123	2,500 V	4.53 × 10^7^ ± 4.33
NRBB57	Glucose	Filtered	Aerobic	3 μg	pNZ123	2,500 V	1.47 × 10^7^ ± 0.90

**TABLE 2:** Primers used in this study.

Target gene	Primer sequence	Amplicon size (bp)
AG- Forward + *Eco*RI	gatcgaattcCGGCGAAGTAACGCTTGATG	546
AG- Reverse + *Hin*dIII	gatcaagcttCCGGATTGGTCAGG	
AD- Forward + *Eco*RI	gatcgaattcGTACCAGAAGGCGTTGGTCA	492
AD- Reverse + *Hin*dIII	gatcaagcttGAAACGCCCTTGATCTTGCC	
PGT— Forward + *Eco*RI	gatcgaattcCACCTACTTCTCCTCTACACC	463
PGT—Reverse + *Hin*dIII	gatcaagcttATCCAACGCTCGATAATAACC	
pFREM-MCS-F	ATAGCACGCCCGCATGCC	Target sequence insertion into pFREM28 insert confirmation.
pFREM-EmR-R	CCGTGTCCGTATGCAGAC	Genome integration confirmation, used with primers below.
AG-ins-com-F	ACCGTCATCCACCACGAATC	721
AD-ins-com-F	GGTCCAGAAGAATCCGGTGG	1,559
EPS-ins-com-F	GTCGGATCGTTGCGGAAATG	1,324

**TABLE 3:** Strains and plasmids used in this study.

Strain	Source	Accession	Study
*E. coli* EC101	*E. coli* JM101 with *repA* from pWV01 integrated in chromosome	VIBV01000001	Law et al. (1995)
*B. breve* UCC2003	Infant isolate, (Breast fed)	CP000303	Mayo et al. (2008)
*B. breve* NRBB01	Infant isolate—provided by Nutricia	CP021384	Bottacini et al. (2018b)
*B. breve* NRBB57	Infant isolate—provided by Nutricia	CP021389	Bottacini et al. (2018b)

**Plasmid**	**Relevant Properties**	**Accession**	**Study**

pNZ44	3 kb; *E. coli/Bifidobacterium* shuttle cloning vector containing constitutive P44 promoter from *L. lactis*; Cm^R^	NA	McGrath et al. (2001)
pNZ123	2.5 kb; *E. coli/Bifidobacterium* shuttle cloning vector; Cm^R^	NA	De Vos (1987), Platteeuw et al. (1994)
pFREM28	2 kb; *B. breve* R-M motif free suicide vector; Em^R^	MT499887	This study

### Phenotypic Screening of Insertional Mutants and Sequence Validation

Single colonies produced following mutagenesis were screened for disrupted phenotype by culturing in mMRS in the presence of control sugar (glucose) or the carbohydrate, of which the corresponding utilization cluster was targeted (raffinose or mannitol) and incubated anaerobically at 37°C. Wild type strains were also assessed for growth on each carbohydrate. Optical density (OD_600nm_) was recorded after 24 h and results were plotted using GraphPad v8.3.0. The gDNA was extracted using GenElute^TM^ Bacterial Genomic DNA Kit (Sigma) following manufacturer’s instructions and quantified by Qubit^TM^ dsDNA BR Assay Kit (Invitrogen). PCR validation of each pFREM28 insertion was performed using bridging primers from within the insertion plasmid and upstream of each target region (Table 2). For the NRBB01 derivative carrying a mutation in the α-galactosidase-encoding gene, a high-quality draft genome was obtained by Illumina Miseq sequencing in order to confirm the expected gene disruption. Reads were assembled using Spades v3.14.0 with kmer lengths of 33, 55, 77, 99, and 127 and contigs were rearranged against the respective reference genome using Mauve v2.4.0. The insert was located using NCBI BLASTn and up to 10 Kb region containing the inserted pFREM28 was extracted from each mutant for further analysis. Open reading frames were predicted with Prodigal in anon mode (-p anon). Functions of protein coding sequences were annotated using BLAST (blastp v2.2.28+) against their respective reference genomes.

## Results and Discussion

### Construction of pFREM28

**FIGURE 1:**
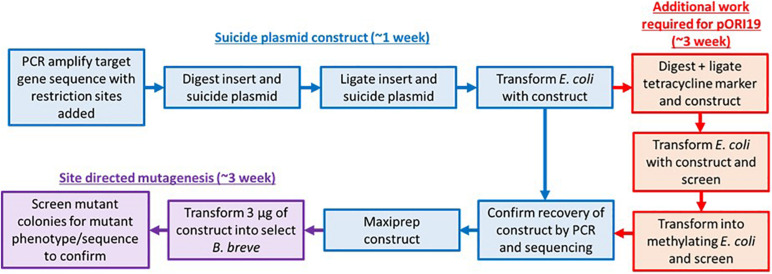
Approaches for site-directed mutagenesis in bifidobacteria. Flow chart describing the original (inclusive of red pathway) and modified steps (exclusion of red pathway which can consist of either methylase cloning or vector methylation) required for performing site-directed mutagenesis in *Bifidobacterium*. The improved pFREM28 vector reduces labor and consumable cost as a step for methylation of the *E. coli-Bifidobacterium* shuttle vector is no longer required.

**FIGURE 2:**
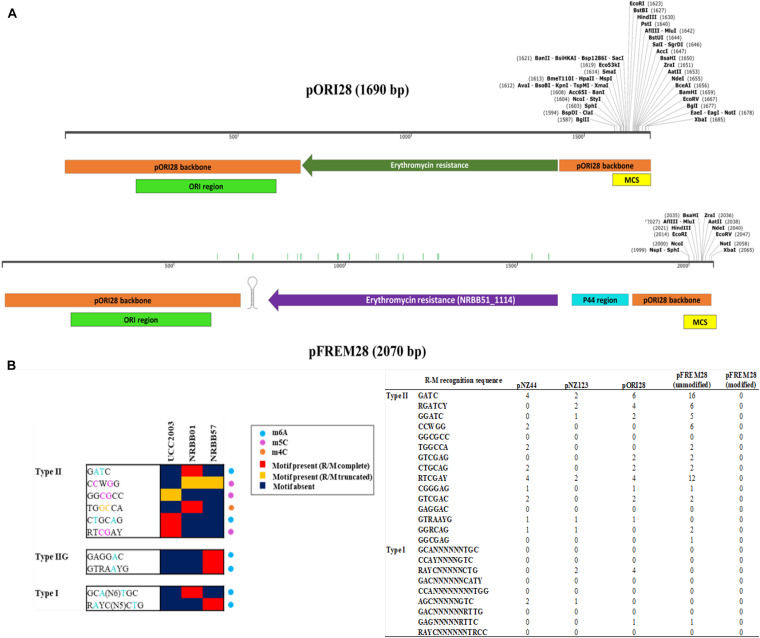
Methylome-directed *in silico* construction of the pFREM28 insertion vector, starting from the pORI28 template. **(A)** Linear plasmid map of the pORI28 original template sequence compared to the newly constructed R-M insensitive vector pFREM28 (Em^R^ Ori + RepA-). The pFREM28 vector contains a different erythromycin resistance gene (locus tag: NRBB51_1114), with the P44 promoter region upstream and a transcriptional terminator downstream. The pORI28 backbone sequence used for the pFREM28 design is indicated in orange, while the ORI region is indicated in green and the multiple cloning site (MCS) in yellow. Green bars along the pFREM28 sequence indicate sites where restriction sites were eliminated. **(B)** Modified extract from Bottacini et al. (2018b) of a heatmap representing presence/absence of R-M motifs within *B. breve* strains. Motifs are grouped based on their assignment to Type I, Type II, and Type IIG R-M system and the type of modification (m6A, m4C, and m5C) is indicated. The number of target sites from the *B. breve* methylome Bottacini et al. (2018b) in plasmids pNZ44, pNZ123, pORI28, pFREM28 (before modification), and pFREM28 (after removal of RM sites) are also indicated. From the data presented it is possible to appreciate how the number of sites and the combination of R-M systems are variable across plasmids and *B. breve* strains.

The Ori^+^ RepA^–^ insertion vector pORI19-tet has been used to date for site-directed mutagenesis in strains of *B. breve*, including *B. breve* UCC2003, JCM 7017, and NCIMB 2258 (O’Connell et al., 2013a; Egan et al., 2014; Bottacini et al., 2018a). For a successful introduction of the vector into a target strain and subsequent integration into the bacterial chromosome by homologous recombination, a methylation step is required in order to protect the vector and cloned insert from the activity of R-M-associated restriction endonucleases in the target strain. Plasmid methylation has so far been achieved *via* the heterologous expression of selected methylases from the target strain into an *E. coli* host (Figure 1 and Supplementary Figure 1), followed by the introduction of the mutagenesis vector in the methylase^+^ *E. coli* host prior to introduction into *B. breve* (O’Connell Motherway et al., 2009). In order to avoid the additional step of vector methylation and to provide a time-effective approach for gene disruption in *B. breve*, we designed an R-M-insensitive synthetic vector pFREM28 (Figure 2A) with the potential for broad application among various *B. breve* strains. This synthetic system was designed using the original pORI28 sequence as a template backbone (see text footnote 1) (Leenhouts et al., 1996), which was subsequently modified *in silico* before synthesis (Figure 2A). A total of 955 bp from the pORI28 vector was used as a backbone, including the ORI region and multiple cloning site. The first step in the design of the novel pFREM28 vector was the replacement of the original erythromycin selection marker in pORI28 with a marker that is suitable for expression in *Bifidobacterium*. In fact, the original erythromycin resistance marker in pORI28 appears to be poorly expressed in *Bifidobacterium*, thus causing unreliable selection. Due to the apparent insufficient expression of the erythromycin resistance gene, current protocols require the introduction of an additional bifidobacterial tetracycline resistance gene in pORI19-tet (a pORI28 derivative), a modification which has proven successful in gene disruption applications of *Bifidobacterium* (O’Connell Motherway et al., 2009; O’Callaghan et al., 2015). The antibiotic resistance marker selected for pFREM28 is a recently identified erythromycin resistance gene from *B. breve* NRBB51 (Bottacini et al., 2018a). The main advantage of using this novel marker is that it confers a high level of erythromycin resistance (up to 256 mg/ml) in *B. breve*, thus ensuring a reliable and clean selection (Bottacini et al., 2018a). The steps undertaken for the construction and optimization of pFREM28 vector are presented in Figure 2. Through *in silico* manipulation the sequence fragment of 929 bp containing the erythromycin resistance gene was extracted from the genome sequence of *B. breve* NRBB51 (locus_tag NRBB51_1114) and introduced to replace the original antibiotic marker in pORI28. In order to ensure a high level of expression of the antibiotic marker, further *in silico* edits were made and a 175 bp sequence containing the constitutive P44 promoter from *L. lactis* was obtained from the relevant publication (van der Vossen et al., 1987) and added to our vector design. The P44 promoter is capable of high expression in both *E. coli* and *Bifidobacterium* hosts and was positioned in our *in silico* construct upstream of the original ribosomal binding site (RBS; 5′-AGGAGC-3′) of the antibiotic marker (the promoter is 40 bp upstream the translational start of the Em^R^ gene). In order to ensure efficient transcriptional termination we decided to add to our *in silico* vector design a rho-independent terminator obtained from a previous transcriptomic study, where we predicted all rho-independent terminators in *B. breve* UCC2003 (Bottacini et al., 2017). The terminator sequence chosen for this purpose 5′-CCCCGACCCCAACCGGTCGGGGCTTCTTGCGTTG-3′ was extracted from the highly expressed *talA/B* operon in *B. breve* UCC2003 (Bottacini et al., 2017) and positioned downstream the Em^R^ gene in our vector sequence design. Finally, in order to make the construct insensitive to the endonuclease activity of known *B. breve* R-M systems, all motifs previously identified as a target sequence of such systems (Bottacini et al., 2018b) were manually removed *in silico* from the vector sequence. The removal of restriction sites was achieved manually by single base substitutions *in silico*, where an alternative synonymous codon was always chosen to avoid any of the target motifs, thus preserving the amino acidic sequence encoded by the gene. A total number of 21 sites of the pFREM28 were thus modified (Supplementary Table 1). The vast majority of these modifications were located in the coding sequence of the erythromycin resistance marker. Notably, no modification was needed to be introduced in the ORI region of the plasmid (base position 210–610), thus not affecting any replication function of the vector. The modifications also necessitated the removal of a number of restriction sites of the multiple cloning site (MCS) region.

Following vector synthesis, the integrity and functionality of the selection marker was confirmed upon self-ligation, transformation and recovery of pFREM28 from the *E. coli* helper strain EC101. This strain, when harboring pFREM28, was shown to be resistant to erythromycin at a concentration of >256 μg/mL, in accord with what described previously in *B. breve* (Bottacini et al., 2018a).

### Improved Transformation Efficiency

**FIGURE 3:**
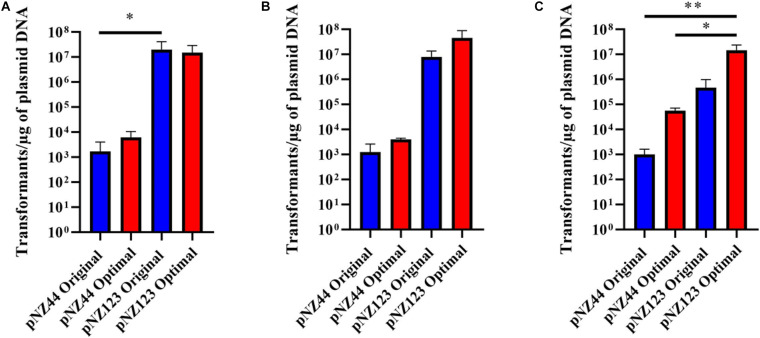
Transformation efficiencies for *B. breve* strains **(A)** UCC2003, **(B)** NRBB01, and **(C)** NRBB57 when “original” (blue) or “optimal” (red) transformation conditions are used for either plasmid pNZ44 or pNZ123. Individual values represent the mean (±SD) produced from triplicate transformations and significance level (Unpaired *t*-test): *p-value ≤ 0.05; **p-value ≤ 0.01. UCC2003 transformation efficiency is significantly higher when comparing pNZ44 to pNZ123 under original conditions. While NRBB01 efficiencies are not significantly different there is a marked increase when pNZ123 is employed. Significant improvements were observed for NRBB57 with pNZ44 under “original” and “optimal” conditions when compared to “optimal” pNZ123. In general, all strains demonstrate slight improvements in transformation efficiency when the “optimal” conditions were used, most notably for NRBB57.

For testing purposes three *B. breve* strains (NRBB01, NRBB57, and UCC2003) were selected from our culture collection, which had previously been predicted to each contain distinct R-M systems (Figure 2B; O’Connell Motherway et al., 2009; Bottacini et al., 2018b). When standard transformation parameters and our routine plasmid pNZ44 were employed with these strains (McGrath et al., 2001), we achieved a transformation efficiency ranging from 10^2^–10^3^ transformants/μg DNA (Table 1 and Figure 3). For successful mutagenesis, transformation efficiencies of at least 10^5^ transformants/μg DNA are recommended (van Pijkeren and Britton, 2012; Zuo et al., 2019). Therefore, in order to improve the transformation efficiency of these strains before attempting targeted mutagenesis we assessed a range of parameters (various carbohydrates for growth, different media and medium preparation methods, changing electroporation voltage parameters, varying plasmid amount, and selecting a plasmid with a smaller number of known R-M motifs) during the preparation and electroporation of the *Bifidobacterium* cells. This fine-tuning of the transformation parameters was performed stepwise in order to increase the transformation efficiency to ∼10^7^ transformants/μg DNA (Table 1 and Figure 3). Strains were grown overnight, and competent cells prepared employing various carbohydrates (glucose, LNnT, lactose, fucose or fucose + lactose), the strains exhibited varied transformation efficiency dependent on the different carbohydrate(s) supplied. The best results for UCC2003 and NRBB57 were observed when glucose was present and fucose + lactose for NRBB01. Secondly, the sterilization treatment of the mMRS medium was modified from autoclaving to filter sterilization (using a 0.22 μM filter), as this resulted in a noticeable increase of the transformation efficiency for NRBB01 and NRBB57 (10^4^ and 10^5^ transformants/μg DNA, respectively).

While certain *Bifidobacterium* species are reasonably aerotolerant with current protocols stating that competent cells can be prepared under aerobic conditions, before anaerobic incubation, we prepared competent cells under anaerobic and aerobic conditions to confirm the exact effect. Upon transformation and plating we did not notice any difference between aerobic or anaerobic prepared cells. However, we decided to proceed with cell preparation under aerobic conditions, as this offered a reduced risk of external contamination by working within a lamina flow hood. The number of R-M motifs present within a given sequence used for transformation are known to have a significant impact on the transformation efficiency (O’Connell Motherway et al., 2009; Bottacini et al., 2018b). We examined our routinely used pNZ44 for R-M motifs known to be present within our strains UCC2003, NRBB01 and NRBB57 (Figure 2B) and identified 19 motifs. To compare we selected the related plasmid pNZ123 (provided by NIZO (De Vos, 1987; Platteeuw et al., 1994) for electrotransformation which had only 12 motifs identified (Figure 2B) and found that indeed there was an increase for each of the strains when an alternative plasmid with fewer R-M motifs was selected. Using pNZ123 we next assayed increasing the amount of plasmid DNA (0.1, 0.2, 0.3, 0.5, 1, and 3 μg plasmid DNA) on the transformation efficiency, the highest amount of plasmid DNA improved the transformation efficiency (∼10^6^ transformants/μg DNA for NRBB57). Finally, the voltage for electroporation was varied (1,500, 1,750, 2,000, 2,250, and 2,500 volts) from the original standard of 2,000 volts and as a result we observed an increase to 10^7^ transformants/μg DNA for all strains when the voltage was increased to 2,500 volts.

While it became apparent that a number of factors are responsible for the transformation efficiency of our strains these were not all uniform. Comparison of the efficiencies achieved with both the “original” and “optimal” conditions (Figure 2) demonstrate that the modifications made had the most profound impact on NRBB57, for both plasmids used. While the other strains did also show improvement, this was marginal compared to NRBB57. Of note, for this strain we obtained the best transformation efficiency with the pNZ123 vector, despite the presence of two RAYC(N5)TGC motifs associated with a Type I RM system. This indicates that the restriction endonuclease of this system is only partially active. In general, we observed that the presence of R-M motifs had the most noticeable effect on the overall transformation efficiency and further justified our approach here to synthetically modify a “common” suicide vector to be free of R-M motifs to improve site-directed mutagenesis. As we did observe an improvement of transformation efficiency for our strains under the “optimal” conditions (Table 1) we determined that these parameters were ideal to proceed with targeted mutagenesis, described below, as we had exceeded the desired threshold of 10^5^ transformants/μg DNA.

### Methylase Free Site-Directed Mutagenesis

**FIGURE 4:**
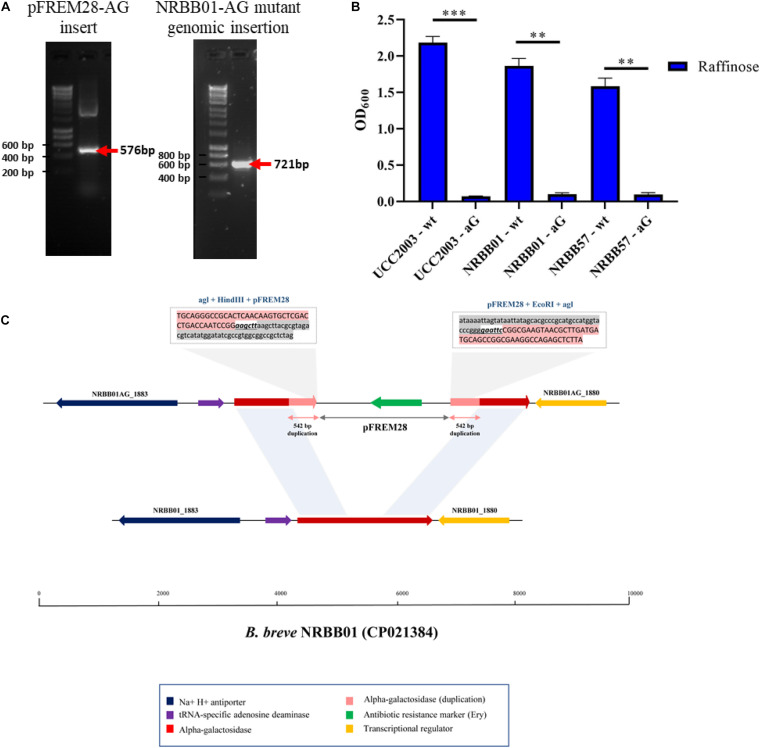
Insertional mutagenesis of the alpha-galactosidase-encoding gene in *B. breve*. **(A)** PCR validation of 542 bp alpha-galactosidase-target gene sequence into pFREM28 and subsequent PCR validation of pFREM28-AG insert into the genome of our *B. breve* strains using primers described in Table 2. **(B)** Optical density (OD600) of *B. breve* strains UCC2003, NRBB01, and NRBB57 wild type and site-directed mutants targeting the alpha-galactosidase-encoding gene were grown for 24 h in the presence of raffinose. Individual values represent the mean (±SD) produced from duplicate cultures and significance level (Unpaired *t*-test): ns, not significant; *p-value ≤ 0.05; **p-value ≤ 0.01; ***p-value ≤ 0.001. **(C)** Sequence confirmation of the integration of the pFREM28-AG vector in the alpha-galactosidase gene of *B. breve* NRBB01. Locus map showing the comparison between *B. breve* NRBB01 wt harboring an intact alpha-galactosidase gene (gene ID NRBB01_1881) and the corresponding locus in the insertional mutant strain containing a disrupted gene. As result of the pFREM28-AG integration (gene ID NRBB01AG_1880 and NRBB01AG_1883) the two flanking regions of the integrant present a 542 bp duplication resulting from homologous recombination.

To assess the ability of pFREM28 to allow gene disruption we targeted two carbohydrate utilization pathways, in addition to a key exopolysaccharide/capsule (EPS) biosynthesis encoding gene previously described in *B. breve* (Fanning et al., 2012; O’Connell et al., 2013b; Bottacini et al., 2014). Our first target was an α-galactosidase gene which had previously been described as involved in the raffinose utilization in *B. breve* UCC2003 (O’Connell et al., 2013b), thus making this gene a suitable candidate to test our insertion system. The genome annotations for NRBB01 and NRRB57 also include a highly homologous α-galactosidase gene (percent identity > 98%, percent coverage 100%) free from any relevant restriction sites (see sites in Figure 2B), as such a 546 bp homologous region was selected and cloned into pFREM28, thus generating a single pFREM28(-AG) construct to be tested in each strain. The subsequent introduction of pFREM28(-AG) in the three tested strains resulted in the successful generation of putative insertion mutants (∼10^1^ Em^R^ transformants/μg DNA) in all three cases (Figures 4A–C), all mutants demonstrated an erythromycin MIC > 256 μg/mL. In each instance of mutant generation, we first screened the mutants *via* PCR to confirm the plasmid insertion (primers used upstream of the insert region and within pFREM28 are described in Table 2 and Figure 4A). The mutants were then phenotypically assessed, validating their expected inability to utilize raffinose (Figure 4B), thus confirming that pFREM28 is capable of successful insertion into the target gene. As a final and definite confirmation of the site-specific integration event in the alpha-galactosidase-encoding gene, a single mutant (NRBB01-AG) was selected to be assessed by Illumina-mediated whole genome sequencing. The sequencing of the strains and subsequent assembly indeed authenticated the interruption of the alpha-galactosidase gene with the pFREM28-AG construct (Figure 4C).

An alcohol dehydrogenase (encoded by the gene with locus tag B7017_1848) has previously been described as correlating with growth on sorbitol/mannitol (Bottacini et al., 2014; Bottacini et al., 2018a) and the finding was confirmed by insertional mutagenesis in *B. breve* JCM 7017 (Bottacini et al., 2014). Also in this case homologous genes (percent identity and coverage 100%) were identified within NRBB01 and NRBB57 (NB the gene appears to be absent in UCC2003) (Bottacini et al., 2018a) and mutants were constructed targeting a 492 bp homologous region, free from any relevant restriction sites (see sites in Figure 2), within the alcohol-dehydrogenase gene for NRBB01 (NRBB01_1667) and NRBB57 (NRBB57_1926). Following transformation, several putative mutants were recovered (∼10^1^ Em^R^ transformants/μg DNA) and validated using primers described in Table 2 (Supplementary Figure 2A), for the two target strains and phenotypic screening confirmed the loss of mannitol utilization ability for NRBB01 and NRBB57 (Supplementary Figure 2B). As a final means of validation, mutants for each strain were selected for Illumina-mediated whole genome sequencing and each confirmed to have had the target alcohol dehydrogenase gene interrupted by pFREM28-AD (Supplementary Figure 2C), thus providing a second confirmation of the effectiveness of our synthetically designed pFREM28 insertion system.

Finally, we targeted the gene encoding the predicted priming undecaprenyl-phosphate galactosephosphotransferase, which had previously been identified in *B. breve* UCC2003 (locus tag Bbr_0430) as a key gene in the EPS biosynthetic pathway (Fanning et al., 2012). *B. breve* NRBB01 is phenotyped as an EPS-producer and contains an annotated priming undecaprenyl-phosphate galactosephosphotransferase (NRBB01_0373) which is 72% identical to that of UCC2003. Therefore, a 463 bp region of NRBB01_0373 was targeted for insertional mutagenesis [NB: NRBB57 is already EPS-negative, while UCC2003 has previously been mutated (Fanning et al., 2012)] free from any relevant restriction sites (see sites in Figure 2). Therefore, we designed a single construct for our validation of EPS biosynthesis in NRBB01 only. Em^R^-resistant transformants were recovered following introduction of plasmid pFREM28-EPS into NRBB01 by electrotransformation and first validated through PCR (see Table 2 and Supplementary Figure 3A). These putative EPS-negative NRBB01 derivatives were also shown to exhibit a clear sedimentation phenotype in liquid growth media [indicative of a lack in EPS production (Fanning et al., 2012)] when compared to the wild type (Supplementary Figure 3B). Illumina-mediated whole genome sequencing once again confirmed our success at generating a site-directed mutant using pFREM28 (Supplementary Figure 3C).

## Conclusion

Genetic manipulation of *Bifidobacterium* has been pursued for a number of years (Missich et al., 1994; Argnani et al., 1996) as a means to better understand their physiology and the mechanism of microbe-host interaction. However, due to the nature of *Bifidobacterium* the introduction of foreign DNA is difficult and only seems to work for selected strains. Their sensitivity to oxygen, thick cell wall and, probably representing the biggest challenge, the diverse and variable R-M systems result in an average transformation efficiency < 10^4^ transformants/μg of DNA (Argnani et al., 1996; Shkoporov et al., 2008; Fukiya et al., 2010; Alvarez-Martin et al., 2012) with a number being completely recalcitrant to transformation. As technology has improved so has our ability to transform various members of the *Bifidobacterium* genus, through the comprehensive predictions of R-M motifs through PacBio sequencing (O’Connell Motherway et al., 2014; Bottacini et al., 2018b) coupled with bisulfite sequencing to identify adenine and cytosine methylation (Darst et al., 2010), targeted methylation of plasmid DNA by chemical method (Yasui et al., 2009; Park et al., 2018), or vectors carrying methylation genes (O’Connell Motherway et al., 2009). Based on our observations, transformation efficiencies can vary from strain to strain in addition to the plasmid selected for transformation. In general, we obtained higher transformation efficiencies (up to 10^7^) with the pNZ123 vector, which contains fewer restriction sites compared to pNZ44. This suggests that pNZ123 is less affected by the R-M barrier in the *B. breve* strains tested. We have also demonstrated here that other factors such as carbohydrate, media preparation, electroporation voltage, and amount of plasmid DNA or type of plasmid can have a significant impact on transformation efficiency.

Current standard lab practice for site-directed mutagenesis involves cloning of one or more parts of a targeted gene in a non-replicating vector that carries an antibiotic selection marker. Additionally, to protect insertional plasmids from R-M system degradation, the construct would need to be first passed through a methylase positive *E. coli* strain which expresses a cloned bifidobacterial methylase. Therefore, we aimed to redesign an insertional plasmid of broad application in *B. breve*, in order to reduce the work necessary to prepare an insertional plasmid before use (Figure 1 and Supplementary Figure 1).

After validating the integrity of pFREM28 and the functionality of the erythromycin selection marker, the vector was successfully employed to target two separate carbohydrate pathways in three *B. breve* strains: UCC2003, NRBB01, and NRBB57 for the alpha-galactosidase gene, while NRBB01 and NRBB57 were manipulated for the alcohol dehydrogenase gene (as UCC2003 lacks of such gene). Finally, we targeted the EPS biosynthesis pathway (NRBB01 only) by inactivating a priming glycosyl transferase gene and observed a “dropping” phenotype typically associated with the lack of EPS production. The advantage of using this modified approach is the reduction of the amount of time and resources required to generate site-directed mutants in members of *B. breve* species without the need for cloning methyltransferases and methylation of the vector prior to transformation. It is worth noting that the removal of the Type I target sites constitutes another advantage of a methylase insensitive vector. According to the recently published *B. breve* R-M systems catalog (O’Connell Motherway et al., 2009; Bottacini et al., 2018b) many *B. breve* strains encode at least one Type I system, with some bifidobacterial strains possess multiple Type I and Type II systems. Methylation of the Type I target sites requires the cloning of not only a methyltransferase gene but also the associated specificity determinants, in order to achieve the desired methylation. Avoiding the cloning of multiple methyltransferases prior to performing insertional mutagenesis on a new strain constitutes another advantage in terms of saving time, resources and labor. Of course one has to take into consideration that the approach presented in the current manuscript has been exclusively applied to members of the *B. breve* species, but a similar approach may be developed for the generation of custom vectors to facilitate the genetic manipulation of even more challenging (bifido)bacterial species. The only foreseeable limitation would be the presence of R-M motifs within the region selected for insertion into pFREM28. If unavoidable a methylation step could be incorporated (as described above). Taken together, our study presents a successful application of the information derived from methylome analysis to extend functional genomics applications in *B. breve* and to provide the technical road map to target other members of *Bifidobacterium*, which have so far proven to be genetically inaccessible. Of course, further testing is required to evaluate the broader application of pFREM28 and similar vectors, including the possibility of employing longer fragments to increase recombination rate. Nevertheless, our study has shown that the design of strain- or species-specific custom vectors is a feasible option and opens the possibility of employing advanced synthetic biology applications to expand functional genomics in *Bifidobacterium*.

## Data Availability Statement

The datasets presented in this study can be found in online repositories. The sequences described in this study have been deposited in GenBank database under the following accession numbers: MT499887, MT978064, MT978066, MT978067, MT978065, and SRA raw data is available at PRJNA662028.

## Author Contributions

EH planned and performed the research and wrote the manuscript. FB performed the *in silico* design and analysis of the pFREM28 vector and assisted in manuscript writing. NC and RB performed research. KL, KA, JK, and JM planned and supervised research. DS planned and supervised research and performed manuscript editing. All authors contributed to the article and approved the submitted version.

## Conflict of Interest

The authors declare that the research was conducted in the absence of any commercial or financial relationships that could be construed as a potential conflict of interest.

## Footnotes

1 https://www.addgene.org/71595

2 https://www.snapgene.com
