# Supplementary material for: Broad Purpose Vector for Site-Directed Insertional Mutagenesis in Bifidobacterium breve
Source: Front Microbiol. 2021 Mar 23;12:636822. doi: 10.3389/fmicb.2021.636822 (PMC8021953; doi:10.3389/fmicb.2021.636822)
Supplement: Supplementary file 1 [file Data_Sheet_1.docx]

**Supplementary material.**

**Table S1.** Base substitution in the design of pFREM28

| **Modification** | **Base position (pFREM28)** | **Original base** | **New base** |
| --- | --- | --- | --- |
| Substitution | 624 | A | G |
| Deletion | 687 | A | - |
| Substitution | 730 | G | T |
| Substitution | 731 | A | T |
| Substitution | 833 | T | G |
| Substitution | 863 | C | T |
| Substitution | 872 | A | G |
| Substitution | 875 | G | A |
| Substitution | 920 | G | A |
| Substitution | 977 | C | G |
| Substitution | 979 | T | G |
| Substitution | 1013 | A | G |
| Substitution | 1091 | C | G |
| Substitution | 1100 | C | G |
| Substitution | 1154 | C | G |
| Substitution | 1172 | T | C |
| Substitution | 1126 | A | G |
| Substitution | 1271 | C | G |
| Substitution | 1274 | A | G |
| Substitution | 1544 | G | A |
| Substitution | 1589 | A | G |
| Deletion | 1834 | T | - |
| Deletion | 1834 | G | - |

**
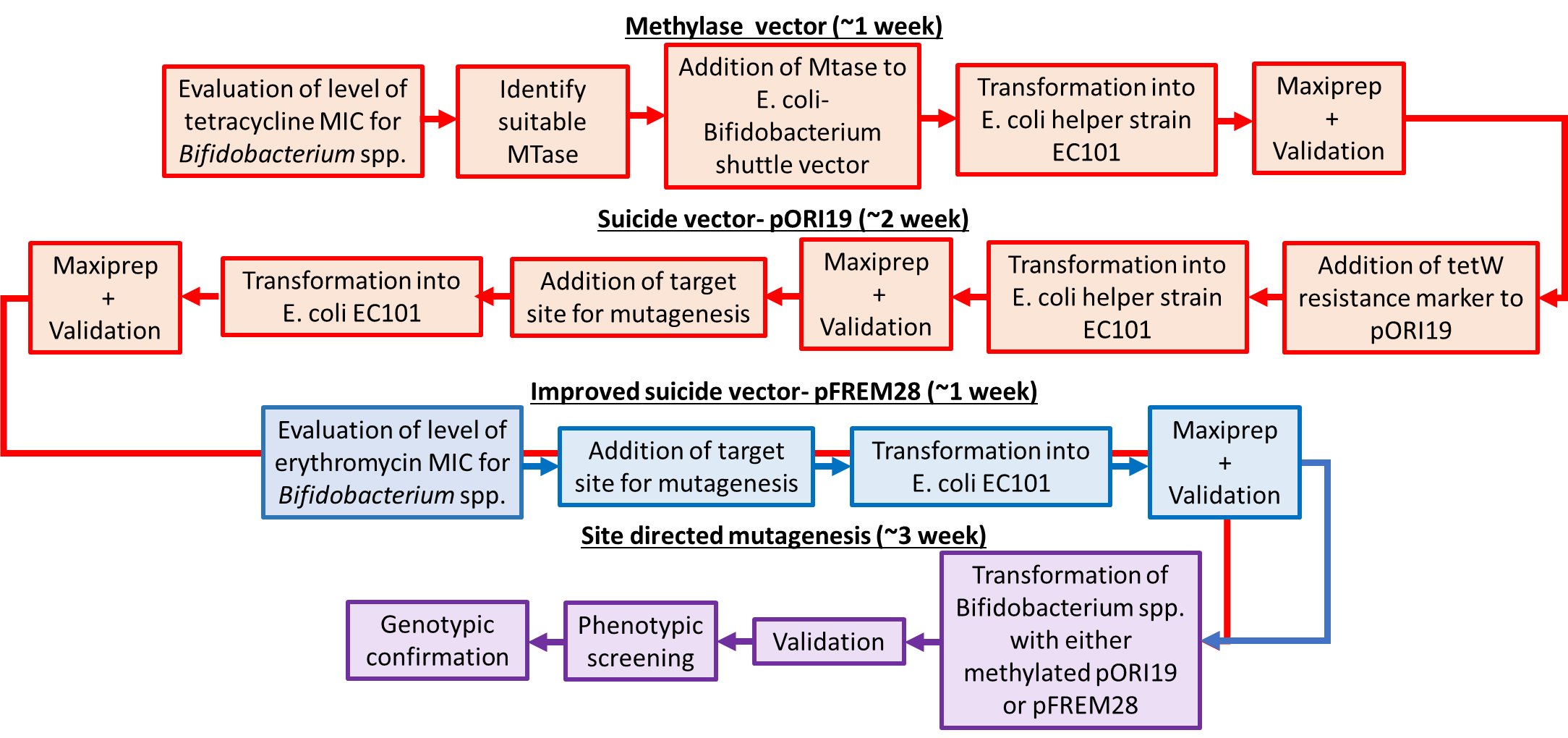
**

**Figure S1.** Detailed approaches for site-directed mutagenesis in bifidobacteria. This flow chart describes the original (red pathway ~3 weeks to complete) and modified (blue pathway details specific steps for pFREM28 utilisation, ~1 week to complete) steps required for performing site-directed mutagenesis in *Bifidobacterium* (purple pathway, ~3 weeks work). The improved pFREM28 vector reduces labour and consumable cost as detailed for this workflow when using previous suicide vector pORI19 a step for methylation of the *E. coli-Bifidobacterium* shuttle vector is no longer required.


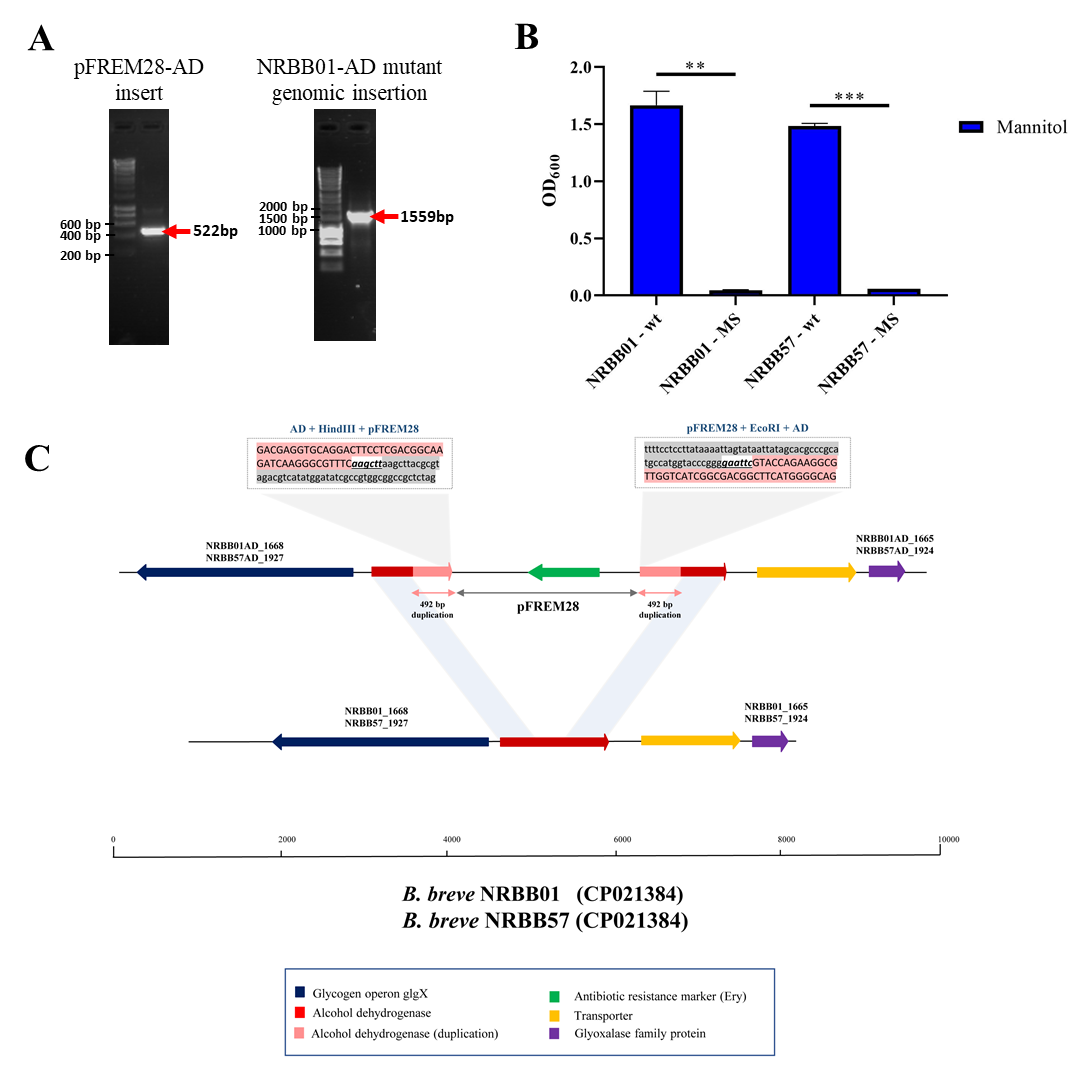


**Figure S2.** Insertional mutagenesis of the alcohol dehydrogenase-encoding gene in *B. breve*. **A)** PCR validation of 492 bp alcohol dehydrogenase-target gene sequence into pFREM28 and subsequent PCR validation of pFREM28-AD insert into the genome of our *B. breve* strains using primers described in Table 3.  **B)** Optical density (OD600) of *B. breve* strains NRBB01 and NRBB57 wild type and site-directed mutants targeting the alcohol dehydrogenase-encoding gene were grown for 24hrs in the presence of mannitol. Individual values represent the mean (±SD) produced from duplicate cultures and significance level (Unpaired t-test): ns, not significant; *, p-value ≤ 0.05; **, p-value ≤ 0.01; ***, p-value ≤ 0.001. **C)** Sequence confirmation of the integration of the pFREM28-AD vector in the alcohol dehydrogenase-encoding gene of *B. breve* NRBB01 and NRBB57. Locus map showing the comparison between *B. breve* NRBB01 wt harbouring an intact alcohol dehydrogenase-encoding gene (gene ID NRBB01_1667 and NRBB57_1926) and the corresponding locus in the insertional mutant strain containing a disrupted gene. As result of the pFREM28-AD integration (gene ID NRBB01AD_1665 and NRBB01AD_1668; NRBB57AD_1924 and NRBB57AD_1927) the two flanking regions of the integrant present a 492 bp duplication resulting from homologous recombination. All mutants had an erythromycin MIC >256 µg/mL.


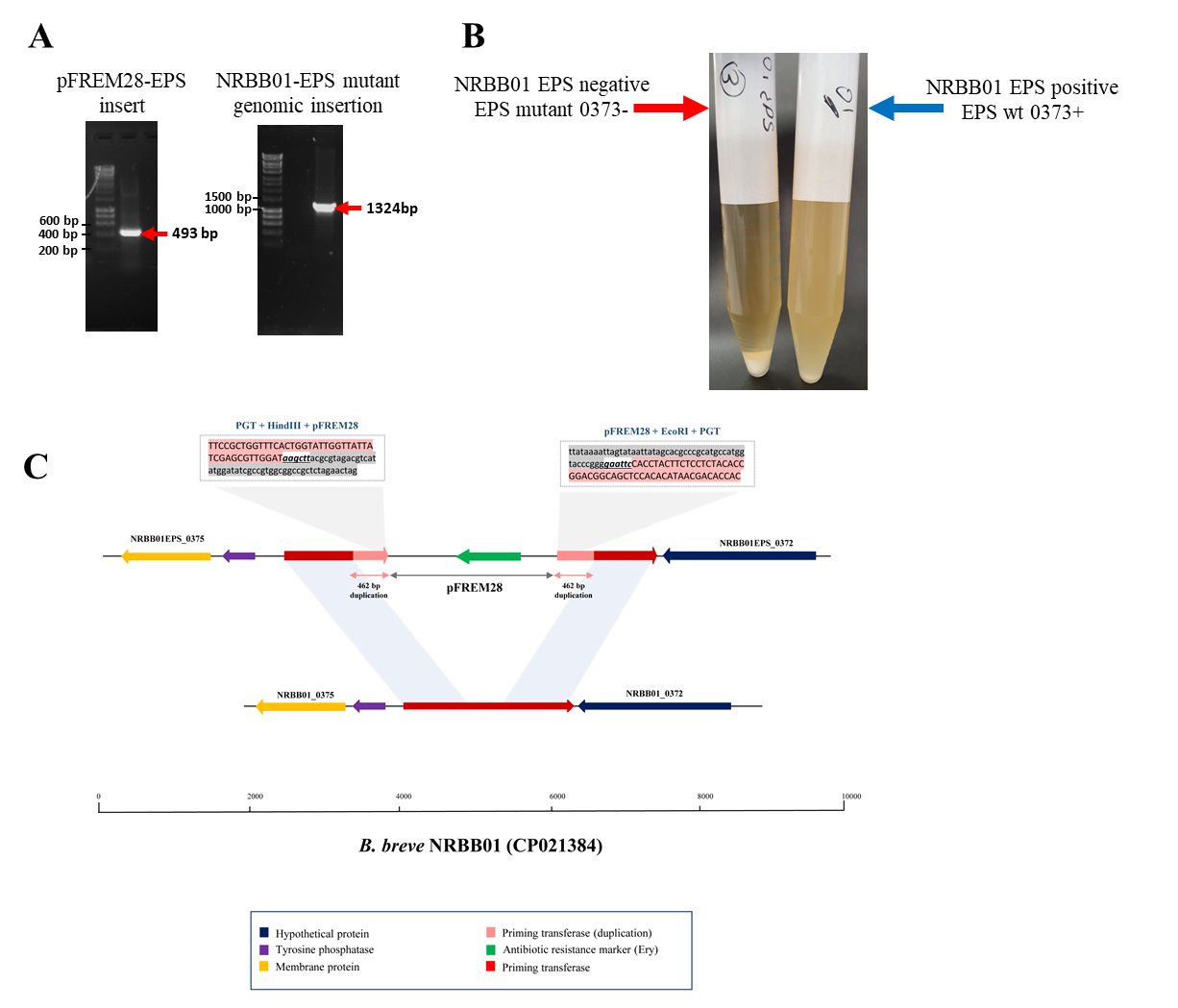


**Figure S3.** Insertional mutagenesis of the undecaprenyl-phosphate galactosephosphotransferase (Und-GalT)-encoding gene in *B. breve*. **A)** PCR validation of 463 bp Und-GalT-target gene sequence into pFREM28 and subsequent PCR validation of pFREM28-EPS insert into the genome of our *B. breve* strain using primers described in Table 3. **B)** NRBB01-EPS mutants compared to wild type NRBB01 demonstrate sedimentation from the media due to the interruption of the Und-GalT-encoding gene (gene ID NRBB01_0373).  **C)** Sequence confirmation of the integration of the pFREM28-EPS vector in the Und-GalT-encoding gene of *B. breve* NRBB01. Locus map showing the comparison between *B. breve* NRBB01 wt harbouring an intact Und-GalT-encoding gene (gene ID NRBB01_0373) and the corresponding locus in the insertional mutant strain containing a disrupted gene. As result of the pFREM28 integration (gene ID NRBB01EPS_372 and NRBB01EPS_375) the two flanking regions of the integrant present a 463 bp duplication resulting from homologous recombination. All mutants had an erythromycin MIC >256 µg/mL.
